# Glycyrrhizin protects against focal cerebral ischemia via inhibition of T cell activity and HMGB1-mediated mechanisms

**DOI:** 10.1186/s12974-016-0705-5

**Published:** 2016-09-08

**Authors:** Xiaoxing Xiong, Lijuan Gu, Yan Wang, Ying Luo, Hongfei Zhang, Jessica Lee, Sheri Krams, Shengmei Zhu, Heng Zhao

**Affiliations:** 1 Department of Anesthesia, The First Affiliated Hospital, School of Medicine, Zhejiang University, Hangzhou, 310003 People’s Republic of China; 2 Department of Neurosurgery and Stanford Stroke Center, Stanford University, 1201 Welch Rd, Stanford, CA 94305 USA; 3 Central Laboratory, Renmin Hospital of Wuhan University, Wuhan, Hubei 430006 People’s Republic of China; 4 Department of Anesthesiology, Zhujiang Hospital of Southern Medical University, Guangzhou, Guangdong 510280 People’s Republic of China; 5 Department of Surgery, Stanford University, Stanford, CA 94305 USA

**Keywords:** Cerebral ischemia, Glycyrrhizin, HMGB1, T cells

## Abstract

**Background:**

Glycyrrhizin (Gly) protects against brain injury induced by stroke. We studied whether Gly achieves its protection by inhibiting T cell activity and high-mobility group box 1 (HMGB1) release in the ischemic brain.

**Methods:**

Stroke was induced by transient middle cerebral artery occlusion in rats and mice. Gly was injected intraperitoneally before or after stroke. We measured infarction, neuroinflammatory cells, gene expressions of interferon-γ (IFNγ), IL-4, and IL-10 in CD4 T cells, HMGB1 release, and T cell proliferation in cultured splenocytes.

**Results:**

Gly treatment reduced infarctions and neuroinflammation characterized by the infiltration of CD68-positive macrophages and myeloperoxidase-positive neutrophils, which corresponds to a reduction in the number of T cells and their subsets, CD4 and CD8 T cells, in the ischemic brain, as measured by flow cytometry. Unlike in wild-type animals, Gly did not offer protection in nude rats and severe combined immunodeficient (SCID) mice who had no T cells, while Gly reduced infarction in both nude rats and SCID mice whose T cells were reconstituted, suggesting that T cells should be the target of Gly. In addition, Gly administration inhibited T cell proliferation stimulated by ConA in in vitro assays and inhibited HMGB1 release from the ischemic brain. Furthermore, Gly attenuated gene expression of IFNγ, but not IL-4 and IL-10 in CD4 T cells. Lastly, HMGB1 promoted T cell proliferation stimulated by ConA, which was inhibited by the addition of Gly.

**Conclusions:**

Gly blocks infarction by inhibiting IFNγ-mediated T cell activity, which is at least partly modulated by HMGB1 activity.

## Background

Glycyrrhizin (Gly), a glycoconjugated triterpene extracted from the licorice root, *Glycyrrhiza glabra* [1], is a natural anti-inflammatory product. It has been used clinically to treat hepatitis B and C in Japan [2]. Gly inhibits pro-inflammatory cytokine and chemokine expression, such as CC-chemokine ligand 2 [3] and TNF-α [4]. More importantly, recent studies have shown that Gly protects brain tissue in global ischemia [5], intracerebral hemorrhage-induced brain injury [6], and focal ischemia [7]. These studies have shown that Gly inhibits high-mobility group box 1 (HMGB1) release, neuroinflammation, and apoptotic cell signaling in the ischemic brain. Despite the solid protective effects of Gly against brain injury, the underlying protective mechanisms remain elusive.

In this current study, we hypothesized that Gly protects against brain injury via mediating T cell activity. This hypothesis is based on two observations. First, T cells have recently been shown to contribute to brain injury, as smaller infarction is found in severe combined immunodeficient (SCID) mice containing no T or B cells [8]. Recently, we have reported that a T cell deficit in nude rats also resulted in smaller infarction induced by middle cerebral artery (MCA) suture occlusion [9]. Second, previous studies have shown that Gly enhances anti-inflammatory cytokines, such as IL-10, and inhibits neutrophil infiltration in lipopolysaccharide-induced acute lung injury and T cell infiltration in liver in concanavalin A (ConA)-induced hepatitis [4, 10]. However, whether T cells are important or not for the protective effect of Gly against stroke has not been studied.

As discussed above, although T cells are well known to contribute to brain injury induced by stroke, how T cells are recruited in the brain and activated after stroke remains unknown. We further hypothesized that T cell activity is modulated by HMGB1, and that Gly inhibits T cell activity via inhibition of HMGB1 release after stroke, based on the following reasons. First, HMGB1 is released in the brain from necrotic neurons as early as 1 h after stroke [11] and is released into the cerebral spinal fluid (CSF) and bloodstream after stroke [12, 13]. Second, once HMGB1 is released into the blood, it may regulate T cell function [14, 15]. Third, Gly has been identified as an HMGB1 inhibitor [16], and Kim et al. reported that Gly attenuated brain injury by blocking inflammation mediated by microglia/macrophages as an HMGB1 inhibitor [7]. Nevertheless, whether HMGB1 regulates T cell activity and whether Gly modulates neuroinflammation via inhibiting HMGB1-mediated T cell activity have not been studied.

In this report, we first examined Gly’s protection in both wild-type rat and mouse stroke models, and then we used T cell-deficient nude rats and SCID mice to study whether T cells are the targets for the protective effects of Gly against stroke. The inhibitive effects of Gly on T cell activities were evaluated using in vitro splenocyte cultures, in which T cell proliferation is induced by ConA stimulation. We also studied the effect of Gly on CD4 T cell functional subsets Th1, Th2, and Treg, by examining gene expressions of IFNγ, IL-4, and IL-10, respectively. We then further examined whether Gly administration inhibited HMGB1 release after stroke and whether HMGB1 promoted T cell activity.

## Methods

### Focal cerebral ischemia

Anesthesia was induced with 5 % isoflurane and maintained by 1.5 to 3 % isoflurane in medical air and balanced with 1 to 20 % O_2_ using a facemask, in Sprague-Dawley (SD) rats (Charles River, Wilmington, MA, USA) and T cell-deficient nude rats (NCI, Frederick, MD, USA) (280 to 320 g) or C57BL/6J wild-type mice (Jackson Laboratory, Bar Harbor, ME, USA) and SCID mice (#001913; B6.CB17-Prkdcscid/SzJ) (25 to 30 g). Rectal temperature was maintained at 37 ± 0.5 °C with a heating pad (Harvard Apparatus, Holliston, MA, USA). Focal ischemia was induced by 100 min of transient MCA suture occlusion in rats or 60 min of occlusion in mice, as described [9, 17, 18]. In brief, we introduced a silicone-coated 4-0 (for rats) or 6-0 (for mice) monofilament (Doccol Corp., Redlands, CA, USA) into the left external carotid artery and advanced through the carotid bifurcation to occlude the MCA. The anesthesia was discontinued after suture insertion. The animals were briefly re-anesthetized after the desired occlusion time, and the filament was withdrawn. Sham-operated animals underwent an identical procedure, except that the suture was not inserted. Physiological measurements were performed as described before [17, 19]. Heart rate, oxygen saturation, and respiratory rate were continuously monitored (STARR Life Sciences Corp., Allison Park, PA, USA). The left femoral artery was cannulated with a polyethylene tube for arterial blood pressure monitoring (Puritan-Bennett Corp., Wilmington, MA, USA). Arterial blood pH, PaO_2_, and PaCO_2_ were monitored with an i-STAT Portable Clinical Analyzer (East Windsor, NJ, USA) on blood drawn from either the femoral or carotid artery.

### Measurement of cerebral infarction area

Infarct size was measured 72 h after stroke. Animals were anesthetized with isoflurane and decapitated, and the brains were removed and cut into five coronal slices with a 2-mm thickness with a rodent brain slicer matrix (Zivic Instruments, Pittsburgh, PA, USA). The sections were stained with 2 % 2,3,5-triphenyltetrazolium chloride (TTC) in saline for 20 min at 37 °C and transferred to a solution of 4 % paraformaldehyde in phosphate-buffered saline (PBS) overnight. The infarct area was analyzed by an observer, blinded to the experimental conditions, using the NIH Image program (ImageJ 1.37v, Wayne Rasband available through the National Institutes of Health) as described previously [9, 17, 18]. To correct for the effects of cerebral edema, the infarction in each section was normalized to the non-ischemic contralateral side and expressed as percentage of the contralateral hemisphere.

### Drug injection

Gly solution (30 mg/ml, #3567800; Calbiochem, San Diego, CA, USA) was prepared in Tris buffer (pH 7.5), and was injected intraperitoneally (i.p.) into animals. To prove the protective effect of Gly against stroke in rats, in the first group, Gly (200 mg/kg) was injected immediately before MCA occlusion and 2 h after reperfusion. In the second group, Gly was injected immediately post-reperfusion and 3.5 h after reperfusion. In the third group, Gly was injected only immediately post-reperfusion. In the control group, the vehicle solution containing no Gly was injected.

### Confocal immunofluorescent staining

Ischemic or sham-operated rats were euthanized with an overdose of isoflurane and perfused with icy PBS, followed by 4 % paraformaldehyde in PBS (pH 7.4), as previously described [9, 17, 18]. The ischemic brains were taken and post-fixed for 48 h in 4 % paraformaldehyde in PBS (pH 7.4) and cut into 50-μm sections. Immunofluorescent staining was carried out on free-floating sections under moderate shaking. All washes and incubations were done in 0.1 M PBS (pH 7.4) containing 0.3 % Triton X-100. The sections were incubated for 1 h with blocking solution (0.1 M PBS, 0.3 % Triton X-100, and 5 % equine serum). After washing, the sections were incubated overnight at 4 °C with primary antibodies.

For double staining of HMGB1 and NeuN proteins, the sections were incubated with a mixture of primary anti-HMGB1 antibody (1:100, ab18256; Abcam Inc, Cambridge, MA, USA) and anti-NeuN antibody (diluted 1:100, MAB377; Millipore, Hayward, CA, USA). Activated microglia/macrophages were stained with mouse anti-rat CD68 antibody (1:200, MCA341GA; AbD Serotec, Oxford, England), and neutrophils were stained with a rabbit anti-human myeloperoxidase (MPO) antibody (diluted 1:50, #A0398; DAKO, Carpinteria, CA, USA). The sections were then washed and incubated for 2 h at room temperature with an Alexa 594-conjugated goat anti-rabbit antibody (1:200 for HMGB1 or MPO) or an Alexa 488-conjugated goat anti-mouse antibody (for CD68 or NeuN, 1:200; Invitrogen, Carlsbad, CA, USA). After washing, the sections were mounted on glass slides using Vectashield mounting medium with 4′,6-diamidino-2-phenylindole (DAPI; Vector Laboratories, Burlingame, CA, USA). A negative control without primary antibodies was performed in parallel. Images were taken with a Zeiss Axiovert inverted fluorescent microscope (Jena, Germany).

To quantify the number of CD68- or MPO-positive cells, three sections (0.20 to 0.70 mm rostral to the bregma) in each animal were randomly chosen, from which the number of CD68- or MPO-positive cells in predefined areas were counted using ImageJ software (NIH) and an average number for each animal was calculated. All counts were performed by an investigator blinded to the coded sections.

### Western blot

The rats were euthanized 72 h after reperfusion with an overdose of isoflurane. CSF was collected and Western blot was performed as described in our previous study [20]. In each lane, 12 μl CSF mixed with 4 μl loading buffer (#NP0007; Invitrogen) was subjected to sodium dodecyl sulfate-polyacrylamide gel electrophoresis using NuPAGE 4 to 12 % Bistris Gel (#NP0323; Invitrogen) for 1.5 h. Protein bands were transferred from the gel to polyvinylidene fluoride membranes (Millipore) for 1 h. After the membranes were blocked with 5 % non-fat dry milk (Bio-Rad Laboratories, Hercules, CA, USA) in PBS/0.05 % Tween-20, a primary anti-HMGB1 antibody was added (diluted 1:500, ab18256; Abcam) and incubated overnight at 4 °C followed by the addition of a horseradish peroxidase-conjugated secondary antibody (1:2000; Cell Signaling Technology, Danvers, MA, USA) for 1 h. The membranes were scanned using Typhoon trio (GE Healthcare, Pittsburgh, PA, USA).

### Serum collection and HMGB1 ELISA

Blood was collected, without anticoagulant, from the rats by cardiac puncture through the right atrium with a syringe and 18-gauge needle. The blood was placed into a 1.5-ml conical tube and left at room temperature for 20 min to allow the blood to clot. Next, the tubes were centrifuged at 5000×*g* for 10 min at room temperature. Afterwards, the serum was transferred to another 1.5-ml conical tube and stored at −80 °C. The serum samples were examined for HMGB1 content using an ELISA kit (# ST51011; IBL International, Hamburg, Germany).

### The effect of Gly on splenocyte proliferation

The [^3^H]-thymidine incorporation assay was used to determine splenocyte proliferation. A total of 2 × 10^5^ cells were seeded in each well of standard 96-well flat-bottom plates with or without 1 μg/ml ConA (Sigma-Aldrich, St. Louis, MO, USA). The cells were incubated for 66 h at 37 °C with 5 % CO_2_. Then, 0.5 μCi [^3^H]-thymidine (Perkin-Elmer, Waltham, MA, USA) was added to each well and incubated for another 18 h. The cells were harvested onto a glass fiber filter. The filter membrane was dried and the amount of radioactivity that corresponds to the number of divided cells during incubation was counted in a β-plate scintillation reader.

To study whether Gly inhibits splenocyte proliferation in vitro, splenocytes were harvested from naïve animals and cultured. A total of 2 × 10^5^ cells were seeded in each well of standard 96-well flat-bottom plates with or without 1 μg/ml ConA. Gly was added to the wells to make final concentrations of 0, 12.5, 25, 50, 100, 200, 400, and 800 μM. The cells were incubated for 66 h at 37 °C with 5 % CO_2_. Then, 0.5 μCi [^3^H]-thymidine was added to each well and incubated for another 18 h. The cells were harvested onto a glass fiber filter. The filter membrane was dried, and the amount of radioactivity that corresponds to the number of divided cells during incubation was counted in a β-plate scintillation reader.

An ex vivo assay was also conducted to analyze the effects of Gly on splenocyte proliferation. Gly (200 mg/kg) was injected twice at 3.5-h intervals into SD rats, who did not receive stroke. The animals were euthanized at 0, 5, 12, 24, 48, and 72 h after the last injection, and splenocytes were prepared for culture. The cells were stimulated with or without ConA, and cell proliferation was measured as described above.

### Cytokine gene expression in functional subsets of CD4 T cells

To detect the functional CD4 subsets (Th1, Th2, and Treg) that are affected by Gly treatment, mice were i.p. injected with Gly (200 mg/kg) at 0 and 3.5 h after reperfusion with 60 min of MCAo. Leukocytes were extracted from the ischemic brain 3 days after stroke, as described above, and then stimulated with Cell Stimulation Cocktail (500X, eBioscience, Cat# 00-4970-93) for 2 1/2 h, to produce IFN-γ, IL-4, and IL-10 gene expressions, which represent the activities of Th1, Th2, and Treg cells, respectively. The cells were washed with FACS buffer and blocked by Fc for 20 min on ice, and then stained by live/dead Aqua (Thermofisher, Cat# L34965), Ly6G (clone: 1A8, Biolegend, Cat#1272639), CD4 (clone: GK1.5, Biolegend, Cat#100421), and CD3 (clone: 17A2, Biolegend, Cat#100205) for 30 min on ice. CD4 T cells were then sorted by FACS sorter into tubes. From these purified CD4 T cells, messenger RNA (mRNA) were extracted using the RNeasy mini kit (QIAGEN, Cat#74104) and reversely transcripted into complementary DNA (cDNA) (Takara, Cat# RR047A). SYBR green master mix (Invitrogen, Cat# 11762-100) was used in real-time PCR. The following primers were used to determine IL-4, IL-10, and IFN-g gene expressions: GAPDH, forward (F): 5′ TTCACCACCATGGAGAAGGC 3′, reverse (R):5′ GGCATGGACTGTGGTCATGA 3′; IL-4, F: 5′ ACAGGAGAAGGGACGCCAT 3′; R: 5′ GAAGCCCTACAGACGAGCTCA 3′; IFNγ, F: 5′ TCAAGTGGCATAGATGTGGAAGAA 3′; R: 5′ TGGCTCTGCAGGATTTTCATG 3′; IL-10, F: 5′ GGTTGCCAAGCCTTATCGGA 3′; R: 5′ ACCTGCTCCACTGCCTTGCT 3′. All the primers used in this study have been validated in a previous study [21].

### The effect of HMGB1 on splenocyte proliferation

To test whether HMGB1 has direct effects on splenocyte proliferation, a total of 2 × 10^5^ cells were seeded in each well of standard 96-well flat-bottom plates. We then added recombined human HMGB1 (rhHMGB1 0, 62.5, 125, 250, 500, 1000 ng/ml) to cultured splenocytes and stimulated them with or without 1 μg/ml ConA. The cells were incubated for 66 h at 37 °C with 5 % CO_2_. The effects of rhHMGB1 on splenocyte proliferation were measured using a Cell Counting Kit-8 (CK-04-11; Dojindo Molecular Technologies, Rockville, MD, USA; *n* = 5), and repeated twice.

To further study the effects of Gly on splenocyte proliferation in the presence of rhHMGB1, splenocytes were cultured and stimulated with ConA, with or without adding rhHMGB1 (500 ng/ml). A series of Gly concentrations of 0, 6.25, 12.5, 25, 50, 100, 200, and 400 μM was added, and then cell proliferations were determined as described above.

### FACS analyses of immune cells in the brains

Mice were deeply anesthetized with isoflurane 72 h after reperfusion. Ischemic ipsilateral brains were dissected after transcardial perfusion with 100 ml of PBS. Brain leukocytes were collected as described previously [22]. In brief, ipsilateral hemispheres were homogenized and filtered through a 70-μm cell strainer. After centrifugation, the cells were resuspended in 7 ml FACS buffer, completely mixed with 3 ml of 90 % Percoll (GE Healthcare), and then 1 ml of 70 % Percoll was loaded under cell suspension. The cell suspension was then centrifuged at 500 g for 30 min at 4 °C. Leukocytes at the interphase were collected for immunostaining and FACS analysis. These cells were stained with differentially fluorochrome-labeled mAbs against TCR, CD4, and CD8 (AbD Serotec) on ice for 30 min to identify T cells, CD4+ T cells, and CD8+ T cells. Data on stained samples were acquired on a BD LSR II flow cytometer using Diva software (v6.1.2; Becton Dickinson, San Jose, CA, USA) and analyzed using FlowJo software (v7.6.2; Tree Star, Ashland, OR, USA).

### Statistical analysis

Data are expressed as mean ± SEM. All data tested met the normal distribution with one-sample Kolmogorov-Smirnov test by using SPSS 16.0. Asymptotic significance for *P* > 0.05 was considered to meet normal distribution. Differences were considered statistically significant for *P* values < 0.05. Student *t* test was used when two-group comparisons were made. Two-way ANOVA was used when both genotype and Gly treatment were taken into account, followed by Bonferroni post-tests using Prism 5 (Software for Science, San Diego, CA, USA).

## Results

### Gly administration reduced infarction in wild-type rats

**Fig. 1:**
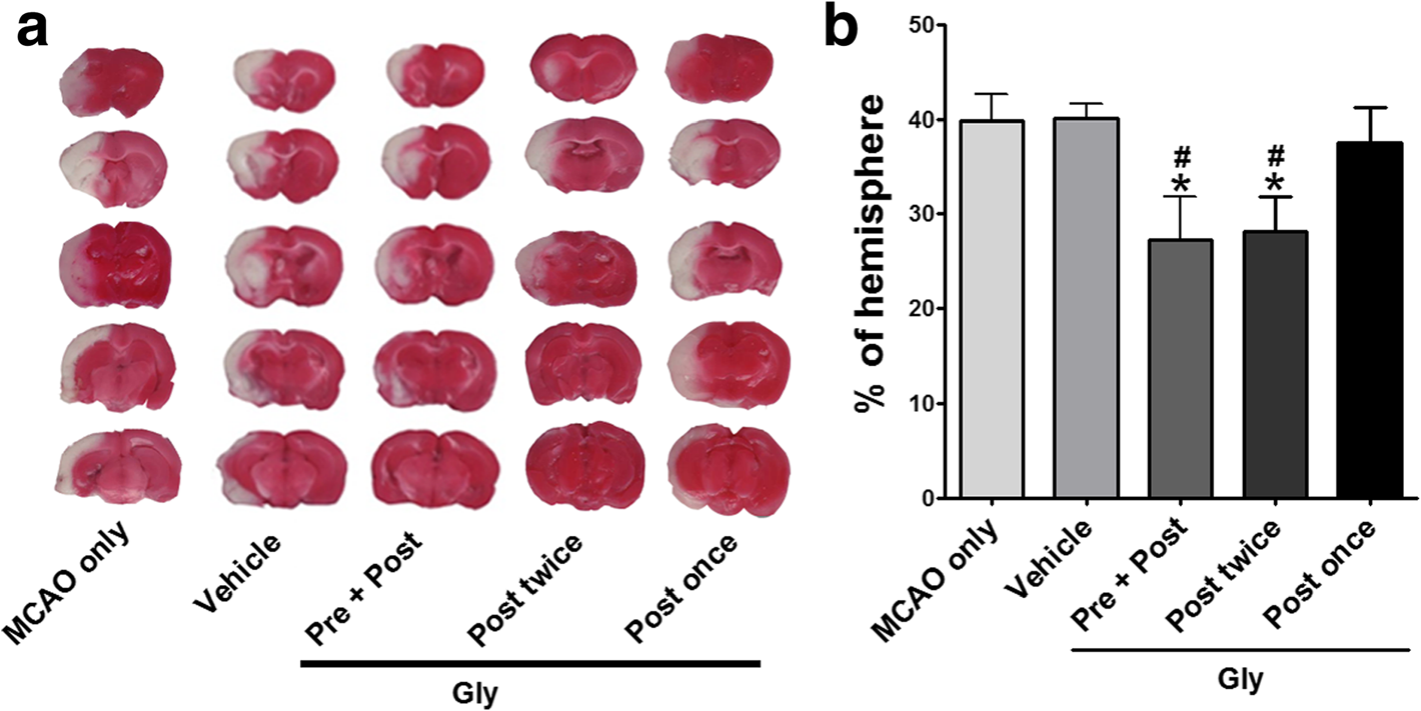
Gly injection reduced infarct size after focal ischemia in wild-type rats. Infarct size in the ipsilateral hemisphere was measured 3 days after stroke onset using TTC staining, then normalized to the contralateral hemisphere and expressed as a percentage. **a** Gly injection reduced infarct size in focal ischemia with 100-min MCA suture occlusion. Representative infarcts stained with TTC are shown. *MCAo only*, the animals received no any treatment; *vehicle*, the stroke animals were injected with vehicle; *Pre + Post*, the drug was injected i.p. immediately before stroke onset and at 2 h; *Post twice*, Gly was injected twice immediately and 3.5 h post-reperfusion; and *Post once*, Gly was injected one time immediately after reperfusion. **b** Bar graphs represent the statistic results. *P < 0.05 vs vehicle; ^#^ *P* < 0.05 vs MCAo only; *n* = 9–13/group

We first examined whether Gly injection reduced infarction induced by transient MCA occlusion in rats. The results showed that infarction was significantly reduced when Gly was injected twice i.p. into the animals, i.e., before MCA occlusion and 2 h after reperfusion (Fig. 1a). We further showed that Gly also resulted in reduction of infarct size when injected twice immediately at reperfusion and 3.5 h after reperfusion (Fig. 1b). Nevertheless, one injection of Gly immediately at reperfusion did not provide protection.

### Gly inhibited neuroinflammation, marked by less expression of CD68 and MPO, and attenuated infiltration of T cells and their subsets

**Fig. 2:**
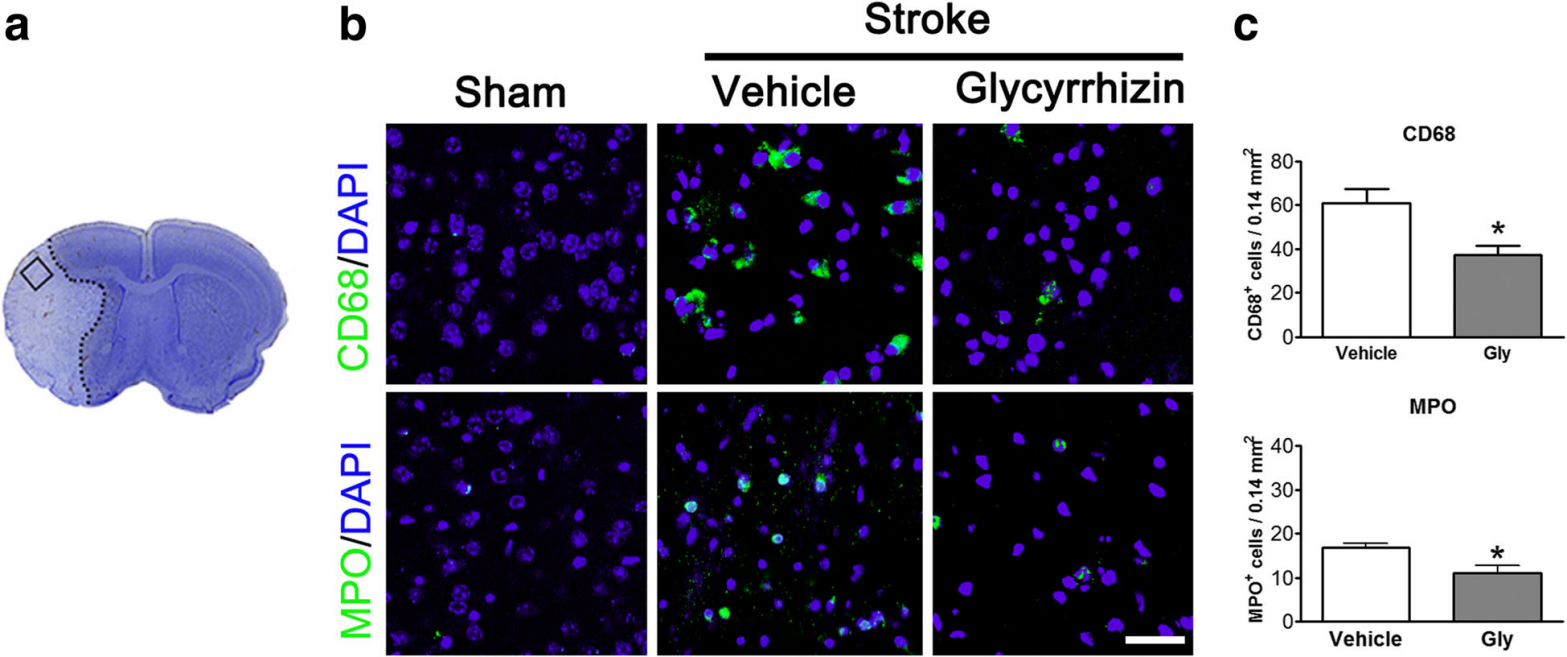
Gly injection attenuated the number of CD68- and MPO-positive cells in the ischemic border. **a** The *left image* shows a representative coronal brain section with cresyl violet staining, on which the *square* represents the area where pictures of immunostaining were taken and cells were counted. **b** Representative immunofluorescence images of CD68 and MPO staining, counterstained with DAPI 72 h after stroke in the ischemic border assessed 72 h after reperfusion. **c** Quantification of CD68- and MPO-positive cells in the ischemic border. *n* = 5/group. *Scale bar* = 50 μm. *P < 0.05 vs vehicle group

Because Gly is known to inhibit neuroinflammation, we then confirmed whether Gly had the same effect in our stroke model. We used confocal microscopy to analyze macrophage and neutrophil activity in the ischemic brains, as macrophages and neutrophils are two of the most important markers for neuroinflammation. The results showed that both CD68 (macrophage marker) and MPO (neutrophil marker) immunostainings were increased in the ischemic brain (Fig. 2) but were attenuated with Gly treatment. The quantification results show that Gly significantly reduced the number of CD68-positive macrophages and MPO-positive neutrophils (Fig. 2).

**Fig. 3:**
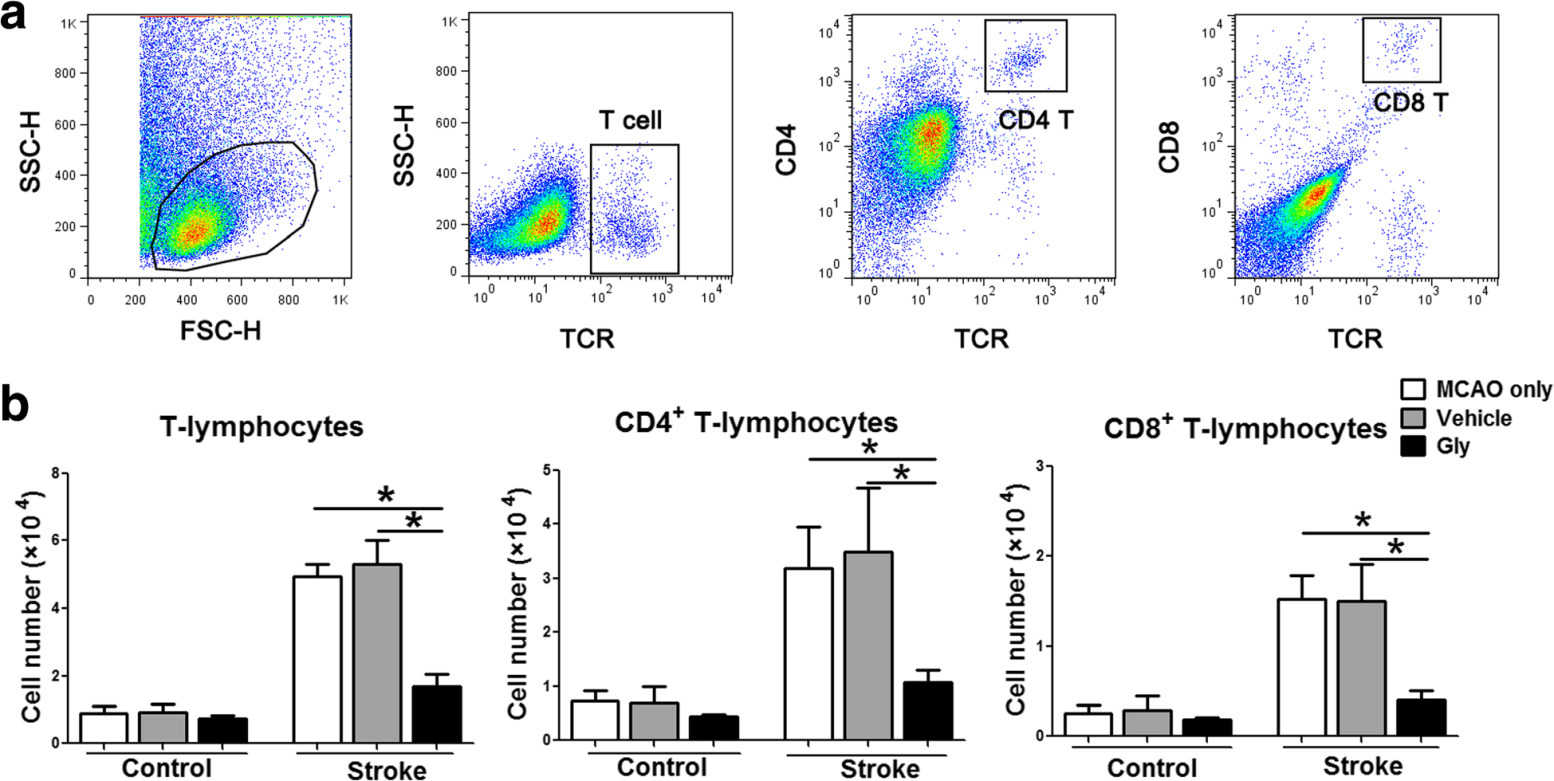
Gly administration blocked T lymphocyte infiltration in the ischemia brain. Mononuclear leukocytes were extracted from ischemic brains 3 days after stroke, and the absolute numbers of lymphocyte subsets were analyzed by FACS. Contralateral hemisphere was collected as control. The drug was injected immediately and 3.5 h after suture removal. **a** Gating strategies for identifying CD4 and CD8 T cells. **b** *Bar graphs* for statistical results of T lymphocytes and T cell subsets. T cells, CD4, and CD8 cells were assessed. *n* = 7 to 12/each group. *P < 0.05 between the two indicated groups

Innate immune cell-mediated inflammation is often related to T cell activity. In addition, we speculated that T cell activity is inhibited by Gly injection. Therefore, we used flow cytometry to quantify T cells and their subsets in the ischemic brains that received Gly treatment. The results showed that the number of T cells and CD4 and CD8 T cells in the ischemic brains was robustly increased without Gly treatment, but was significantly inhibited by Gly administration (Fig. 3). Therefore, the protective effects of Gly are associated with its ability to inhibit T cell infiltration of the ischemic brain.

### T cells are the targets for the protective effect of Gly against stroke

**Fig. 4:**
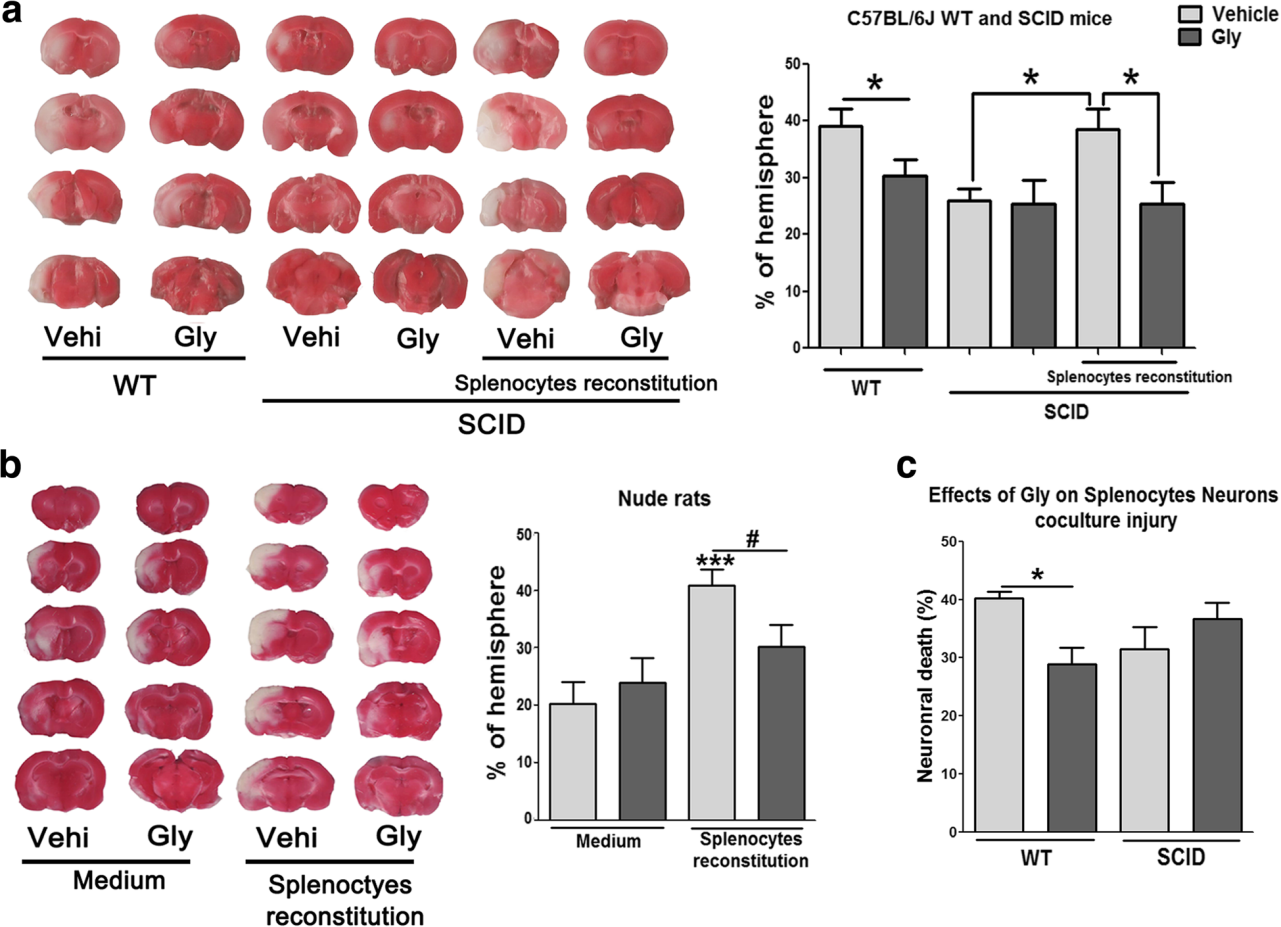
Gly injection did not reduce infarction in SCID mice and T cell-deficient nude rats. Infarct size in the ipsilateral hemisphere was measured 3 days after stroke onset using TTC staining, then normalized to the contralateral hemisphere and expressed as a percentage. **a** The effects of Gly injection on brain infarction in SCID mice with and without T cell reconstitution. *n* = 7 to 9/each group. *P < 0.05 between the two indicated groups. **b** Gly treatment did not reduce infarct size in focal ischemia with 100 min MCA suture occlusion in nude rats. The drug was injected i.p. immediately before stroke onset and 2 h after reperfusion. The animals were divided into four groups. Nude rats in the first two groups were injected with vehicle and Gly, while nude rats in the second two groups were reconstituted with wild-type splenocytes, thus T cells were reconstituted. ***P < 0.001 vs control ischemia with vehicle injection; ^#^ *P* < 0.05 between the two indicated groups. **c** The effects of Gly on neuronal death in the co-culture with splenocytes. Neurons were co-cultured with wild-type (WT) and SCID splenocytes. *P < 0.05 between the two indicated groups

We then test if T cells are the targets for the protective effects of Gly. As STAIR requires that multiple animal models should be used to confirm a drug’s protective effects against stroke, both mice and rats were used. We first examined whether Gly injection reduces infarction in SCID mice in which no T cells or B cells are present. The results showed that Gly injection significantly inhibited infarction in the wild-type mice but showed no protection in the SCID mice. Nevertheless, when T cells and B cells were reconstituted in SCID mice, Gly injection again reduced infarct sizes (Fig. 4a). These results suggest that T cells might be the essential target for the protective effect of Gly.

In addition, we show that infarction was not altered by Gly in the nude rats (Fig. 4b), suggesting again that T cells may be the targets of Gly treatment. We then reconstituted T cell populations in the nude rats by injection of splenocytes from wild-type rats. The results show that reconstitution of T cells resulted in larger brain infarction, which was significantly inhibited by Gly treatment (Fig. 4b).

We previously established an in vitro co-culture model of lymphocytes and neurons, which shows that the presence of T cells results in neuronal injury [18]. We used this model to test if Gly inhibited neuronal death mediated by T cells. The results showed that the co-culture of wild-type splenocytes with neurons resulted in 40 % neuronal death. This was significantly inhibited by the addition of Gly to the culture (Fig. 4c). Nevertheless, the co-culture of splenocytes without T cells resulted in less neuronal injury, and this was not altered by the addition of Gly. This experiment further suggests that T cells are the target of Gly administration.

### Gly inhibited T cell proliferation

**Fig. 5:**
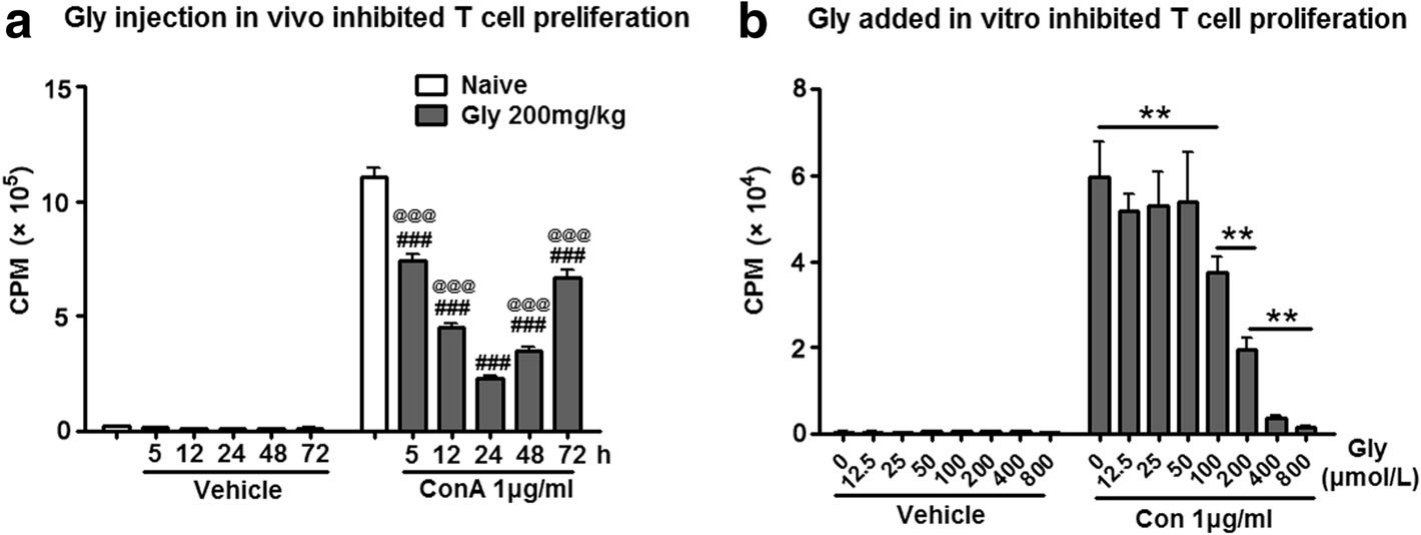
Effect of Gly injection on T cell proliferation. **a** An ex vivo assay suggests that T cell proliferation was inhibited in splenocytes harvested from animals injected with Gly. The drug was injected twice in rats at an interval of 3.5 h. No stroke or sham surgery was performed. Splenocytes were harvested 5, 12, 24, 48, and 72 h after the last drug injection, cultured, and stimulated with ConA. Proliferation was assessed by measuring cell numbers taken by the [^3^H]-thymidine incorporation assay. ^###^ *P* < 0.001 vs naïve; ^@@@^ *P* < 0.001 vs 24 h with ConA injection. **b** In vitro assay shows that Gly dose-dependently inhibited T cell proliferation stimulated by ConA. Splenocytes (2 × 10^5^) harvested from naïve rats were cultured and stimulated with ConA and [^3^H]-thymidine, and different dosages of Gly were added. T cell proliferation was then measured. *CPM* counts per minute, which is the counted radioactivity. **P < 0.01 between the two indicated groups

If T cells are critical for the protective effects of Gly, T cell activity should be inhibited by Gly. We therefore examined whether Gly inhibits T cell proliferation in two sets of experiments. In the first experiment, splenocytes harvested from naïve rats were cultured and stimulated with ConA, which is known to promote T cell proliferation. A series of dosages of Gly was added to the cultures. T cell proliferation was assessed, measuring cell numbers taken by the [^3^H]-thymidine incorporation assay. The results suggest that Gly dose-dependently blocked T cell proliferation (Fig. 5b).

In the second experiment, Gly was injected into rats who received no further treatment, sham surgery, or stroke. The splenocytes were harvested at 5, 12, 24, 48, and 72 h after injection, cultured, and stimulated with ConA. The results showed that Gly treatment inhibited T cell proliferation at all time points, but with the strongest inhibition at 24 h (Fig. 5a).

### Gly inhibited Th1 cytokine gene expression

**Fig. 6:**
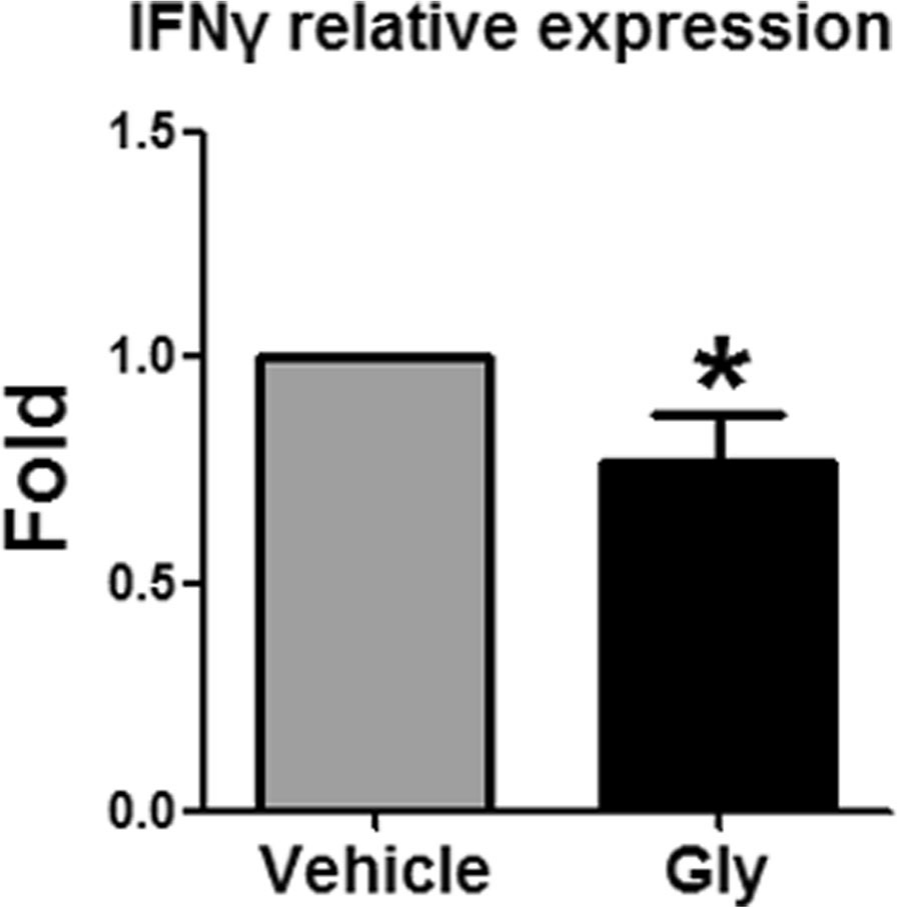
Gly inhibits IFNγ gene expression in CD4 T cells. Leukocytes were isolated from the ischemic brain 3 days after stroke, and CD4 T cells were purified by using FACS sorter. Gene expression of IFNγ, IL-4, and IL-10 were measured by RT-qPCR. However, IL-4 and IL-10 gene expressions were not detectable. Gene expression of IFNγ was significantly inhibited by Gly administration. *N* = 3, *P < 0.05 vs control stroke

CD4 T cells were sorted from ischemic brains based on CD3^+^CD4^+^ cells, and gene expressions of IFNγ, IL-4, and IL-10 were measured by RT-qPCR to detect Th1, Th2, and Treg cell response with Gly administration. In the sham group without stroke, few CD4 cells could be detected (data not shown); thus, no CD4 T cells from the sham group could be used for gene expression measurements. For CD4 T cells isolated from the ischemic brain, gene expressions of IL-4 and IL-10 could barely be detected (data not shown), but gene expressions of IFNγ was relatively high, which was significantly inhibited by Gly administration (Fig. 6).

### T cell activity was promoted by HMGB1 but was inhibited by Gly administration

**Fig. 7:**
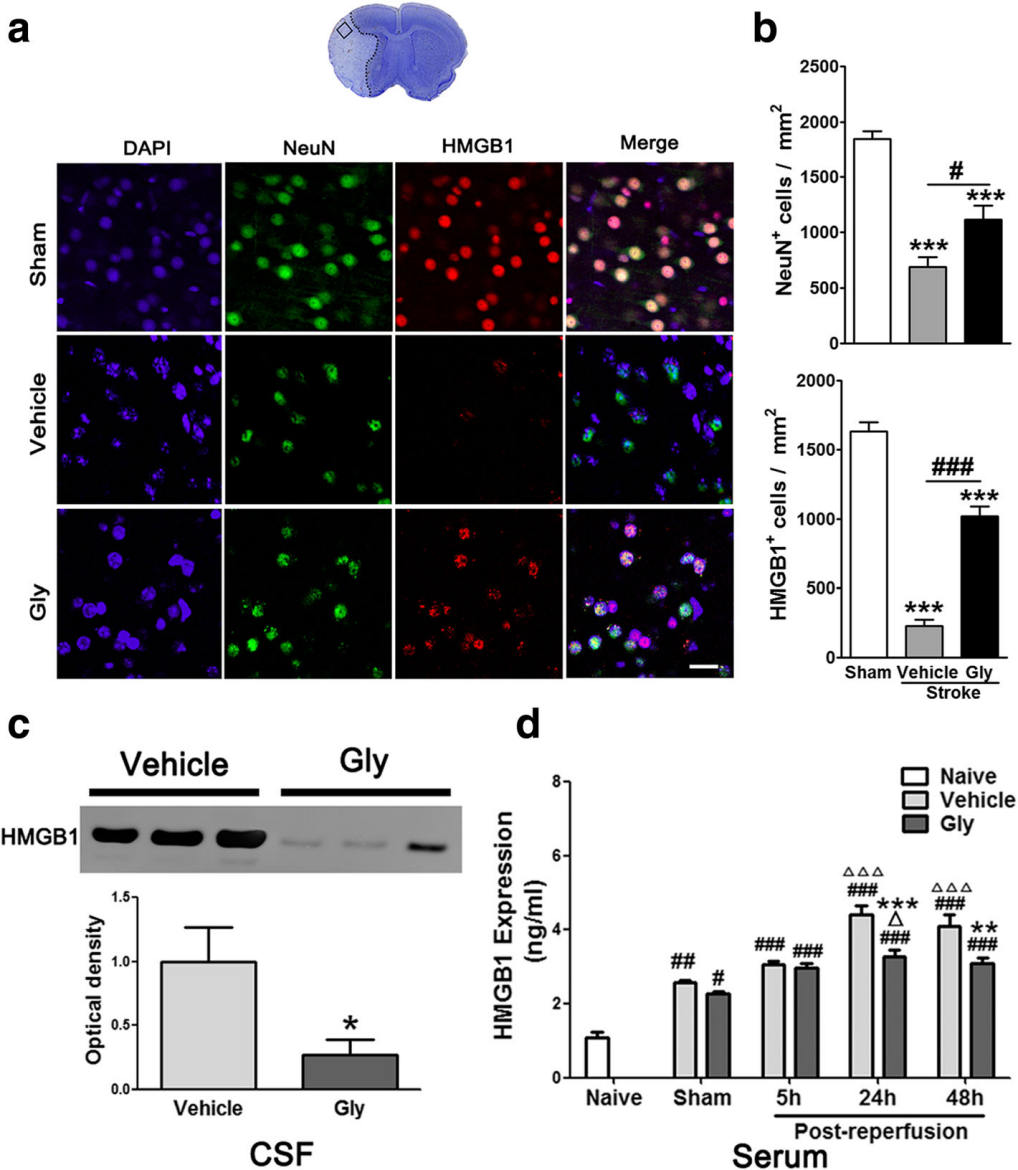
Gly inhibits HMGB1 release in ischemic area and HMGB1 expression in CSF and serum. **a** Representative imaging of double immunofluorescent labeling for HMGB1 with NeuN in the ischemic brain at 3 days after stroke. The *top image* shows a representative coronal brain section with cresyl violet staining, on which the *square* represents the area where pictures of immunostaining were taken. *Scale bar* = 50 μm. NeuN-positive cells are *green*, HMGB1-positive cells are *red*, and merged cells are *yellow*. **b** *Bar graphs* show the statistical results of NeuN- and HMGB1-positive cell numbers. ***P < 0.001 vs sham; ^#^ *P* < 0.05, ^###^ *P* < 0.001, respectively, between the two indicated groups. *n* = 5/group. **c** Western blotting confirmed that HMGB1 release in the CSF was inhibited by Gly. Representative protein bands of HMGB1 are shown, and the *bar graph* shows statistical results. The drug was injected immediately before and 2 h post-reperfusion after 100 min of MCA suture occlusion. CSF was collected 24 h later. No protein bands were detected in the samples from sham surgery (data not shown). *P < 0.05 vs vehicle. *n* = 5/group. **d** Gly injection inhibited HMGB1 release in the serum in rats with MCA suture occlusion. HMGB1 levels in serum after 5, 24, and 48 h of reperfusion were assessed by ELISA. ^#^ *P* < 0.05, ^##^ *P* < 0.01, ^###^ *P* < 0.001 vs naïve, respectively; ^Δ^ *P* < 0.05, ^ΔΔΔ^ *P* < 0.001 vs sham, respectively; **P < 0.05, ***P < 0.001 vs vehicle at the same time point, respectively

We speculated that T cell activity is directly related to HMGB1 release after stroke and that Gly inhibited T cell activity via inhibition of HMGB1 release. To prove this, we first examined HMGB1 expression in the ischemic brain 3 days after stroke. The confocal microscopy results suggest a strong immunostaining of HMGB1 in the nuclei of neurons in sham animals, but its expression was robustly reduced after stroke, suggesting that HMGB1 might have been released (Fig. 7a). Nevertheless, Gly administration improved HMGB1 staining in the nuclei (Fig. 7a), suggesting HMGB1 release was inhibited. The statistical results suggest that Gly increased the numbers of both HMGB1- and NeuN-positive cells (Fig. 7b). Second, we used Western blotting to examine protein levels of HMGB1 in the CSF collected 3 days post-stroke, and the results show that HMGB1 levels were robustly increased in the CSF after stroke but were significantly blocked by Gly (Fig. 7c). No protein bands of Gly were detected in the CSF from animals with sham surgery (data not shown). Third, we further analyzed HMGB1 levels in the serum by ELISA. HMGB1 protein levels were increased in the serum from 5 to 24 to 48 h after stroke, and Gly significantly blocked increases in HMGB1 at 24 and 48 h (Fig. 7d). Taken together, Gly administration inhibited HMGB1 release from the ischemic brain after stroke.

**Fig. 8:**
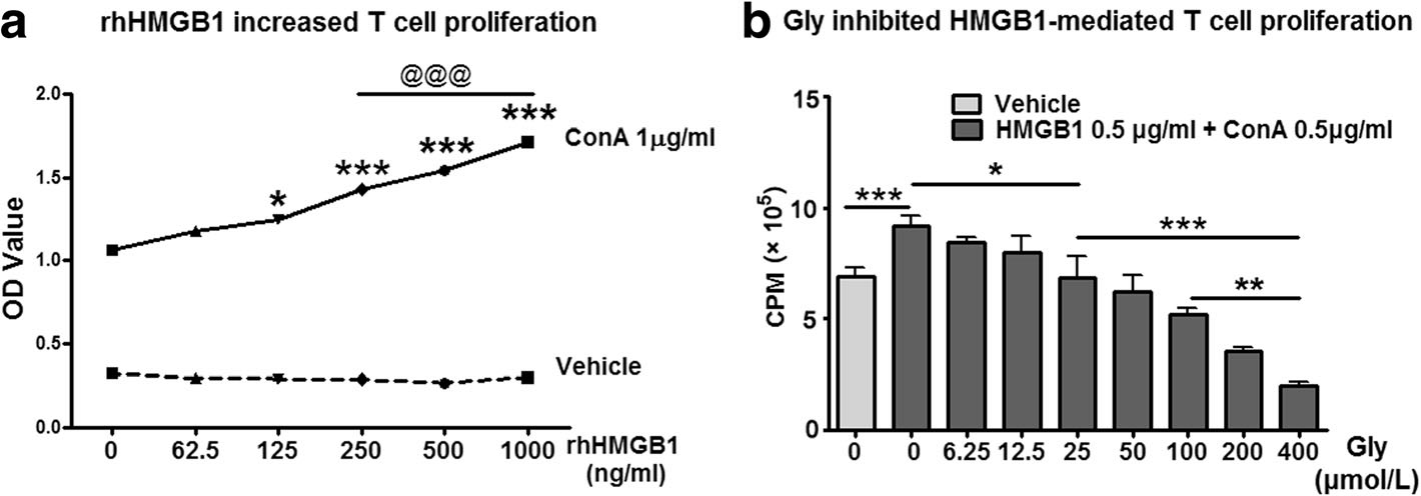
The effect of Gly on rhHMGB1-mediated T cell proliferation in cultured splenocytes. **a** rhHMGB1 protein promoted splenocyte proliferation in vitro. Splenocytes harvested from naïve rats were cultured, and rhHMGB1 was added in a series of concentrations. The cell numbers were detected using Cell Counting Kit-8. The results suggest that rhHMGB1 dose-dependently increased splenocyte proliferation. *P < 0.05, ***P < 0.001 vs 0 ng/ml of rhHMGB1, respectively. ^@@@^ *P* < 0.001 between the two indicated groups. **b** The addition of Gly inhibited rhHMGB1-mediated T cell proliferation. Different concentrations of Gly were added to the cultured splenocytes with rhHMGB1 and ConA, and T cell proliferation ratios were measured. *P < 0.05, **P < 0.01, ***P < 0.001, respectively, between the two indicated groups. *n* = 5/group. *OD* optical density, *CPM* counts per minute

We then examined the hypothesis that HMGB1 promotes T cell activation. Indeed, when rhHMGB1 protein was added to a cell culture of splenocytes, it dose-dependently promoted T cell proliferation stimulated by ConA (Fig. 8a), suggesting that HMGB1 is a direct effector on T cell function. We then examined the effect of Gly on HMGB1-mediated T cell proliferation. T cell proliferation stimulated by ConA in the presence of HMGB1 was evaluated, and we showed that HMGB1 promoted T cell proliferation but that proliferation was inhibited by Gly in a dose-dependent pattern (Fig. 8b).

## Discussion

Although a few studies have shown that Gly has solid neuroprotective effects against cerebral ischemia, the underlying protective mechanisms remain elusive. In this study, after confirming the protective effects of Gly in both rats and mice, we report for the first time that Gly reduces brain infarction by inhibiting T cell activity associated with HMGB1 release. This is supported by several lines of evidence. First, Gly inhibited infarction in both wild-type rats and mice, but not in nude rats and SCID mice. This was further confirmed by the protective effects of Gly in an in vitro co-culture of splenocytes and neurons, suggesting that T cells are the targets of Gly protection against stroke. Second, T cell proliferation was inhibited when Gly was injected in vivo and when Gly was added to cultured splenocytes, as evaluated by an in vitro assay. Third, Gly administration inhibited gene expressions of IFNγ, but not IL-4 and IL-10 in purified CD4 T cells. Fourth, Gly injection in vivo inhibited HMGB1 release in CSF and serum after stroke, and an in vitro assay showed that Gly inhibited T cell proliferation stimulated by HMGB1. Taken together, it is plausible that Gly inhibits brain injury by inhibiting T cell activity, which is at least partly mediated by HMGB1 release after stroke.

A few recent studies have reported that Gly protects against brain injury induced by cerebral ischemia [23–27]. Gly inhibits pro-inflammatory responses, including microglial activation [7], TLR cell signaling pathways [23], pro-inflammatory cytokines, free radical enzymes, and transcription factors, such as IL-1, TNF-α, COX-2, iNOS, NF-kB, and HMGB1 release [23–27]. Despite these studies, whether T cells are directly involved in the protective effect of Gly against stroke has not been studied. T cells have been demonstrated to contribute to brain injury induced by stroke in mice [8, 28–30]. T cells, like macrophages and neutrophils, were found to infiltrate the ischemic brain after stroke [31], suggesting that T cells may form physical contact with brain tissue or secrete inflammatory factors to destroy neurons. In addition, infarct sizes were robustly reduced in immunodeficient SCID mice lacking both T cells and B cells and that with the reconstitution of T cells, but not of B cells, increased infarction [8]. More recent studies suggest that deficiency in either CD4 or CD8 T cells results in smaller infarct size [28, 29], while deficiency in regulatory T cells (Treg) leads to enlarged, delayed infarct sizes [30]. Our laboratory not only confirmed the effects of T cells and CD4 and CD8 T cells, but also further showed the distinctive roles of CD4 T cell subtypes in mice, with reduced infarction in Th1-deficient mice and increased infarction in Th2-deficient mice, while Treg deficiency had no effects on acute infarction [18]. Despite different T cell subsets having distinct effects, total T cells contribute to brain injury.

For the first time, we provide solid evidence that T cells are the target of the protective effect of Gly against stroke. Initially, we found that the robust protective effect of Gly on infarction and inflammation is related to the reduced number of infiltrating T cells and CD4 and CD8 T cells, suggesting that T cells may be one of the targets of Gly administration. Indeed, we found that Gly had no protective effect in nude rats and SCID mice who are T cell-deficient, but that Gly gained a protective effect in these animals when T cells were reconstituted. Similar to these in vivo results, wild-type splenocytes co-cultured with neurons resulted in more neuronal death than SCID splenocytes; Gly attenuated neuronal death in this co-culture, but it had no protection in neurons co-cultured with T cell-deficient splenocytes. Thus, both in vivo and in vitro data indicate that the presence of T cells is essential for the protective effect of Gly. Furthermore, we provided direct evidence that Gly blocks T cell proliferation. An ex vivo assay showed that T cell proliferation in cultured splenocytes harvested from animals treated with Gly was inhibited compared with vehicle injection. In vitro assays showed that the addition of Gly to splenocyte cultures also blocked T cell proliferation. Lastly, we showed that Gly administration inhibited IFNγ gene expression, but not IL-4, and IL-10 gene expression in purified CD4 T cells. Thus, it is likely that Gly inhibited Th1 functional subsets, but not Th2 and Treg cells. Taken together, it is plausible that Gly blocks brain injury by inhibiting T cell functions.

Although T cells are involved in brain injury, how they are recruited and activated after stroke remains unknown. We provide evidence that T cell activity can be induced by HMGB1, as rhHMGB1 promoted T cell proliferation in cultured splenocytes. We further prove that Gly inhibited HMGB1-modulated T cell proliferation in cultured splenocytes, suggesting the protective effects of Gly are at least partly dependent on their inhibitive effects on HMGB1-mediated T cell functions. Our in vivo studies further show that Gly attenuated HMGB1 release from the ischemic brain and attenuated its levels in CSF and serum. Taken together, inhibition of HMGB1 release and T cell function may be a major reason for the protective effects of Gly against stroke.

Our central finding in this study is that Gly reduces brain infarction by inhibiting T cell function, which has significant values for clinical translation. Most neuroprotectants in previous studies were designed to target pathological cascades occurring in the ischemic brain, such as glutamate antagonist, free radical scavengers, and apoptotic and necrotic cell signaling pathways. However, our current study suggests that T cells in the peripheral circulation are a therapeutic target for Gly administration. This provides a rationale to facilitate its clinical translation.

There are some limitations in this study. In Fig. 4, nude rats and SCID mice were reconstituted with splenocytes but not purified T cells. Thus, a question remains, whether other transferred cells, such as monocytes/macrophages, and NK cells, have contributed to the protective effect of Gly treatment. Indeed, we cannot exclude a possibility that Gly also inhibits brain injury by blocking macrophage activities, as our additional preliminary studies showed that Gly had a trend of inhibiting macrophage activities (data not shown). It is known that T cells cross talk with macrophages, whether Gly blocks brain injury by inhibiting both T cells and macrophages will be studied in the future.

## Conclusions

In summary, we report that Gly is neuroprotective in wild-type but not in T cell-deficient animals. It is likely the protective effects are achieved by its inhibitive effects on T cell activity and at least partly through its ability to block HMGB1-mediated T cell function. We define an alternative approach to treating brain injury induced by stroke by targeting circulating T cells.

## Abbreviations

ConA: Concanavalin A
CSF: Cerebral spinal fluid
DAPI: 4′,6-Diamidino-2-phenylindole
Gly: Glycyrrhizin
HMGB1: High-mobility group box 1
MCA: Middle cerebral artery
MPO: Myeloperoxidase
PBS: Phosphate-buffered saline
rhHMGB1: Recombined human high-mobility group box 1
SCID: Severe combined immunodeficient
SD: Sprague-Dawley
Treg: Regulatory T cells
TTC: 2,3,5-Triphenyltetrazolium chloride
